# Effects of Timing of Microbial Exposure on Microbiome Assembly and Amphibian Immune Development

**DOI:** 10.1002/ece3.74063

**Published:** 2026-08-02

**Authors:** Abigail J. Miller, Myung Chul Jo, Cassandra K. Hui, Juli Petereit, Douglas C. Woodhams, Jamie Voyles

**Affiliations:** ^1^ Department of Biology University of Nevada, Reno Reno Nevada USA; ^2^ Environmental Health and Safety University of Nevada, Reno Reno Nevada USA; ^3^ Nevada Bioinformatics Center (RRID:SCR_017802), University of Nevada, Reno Reno Nevada USA; ^4^ Department of Biology University of Massachusetts Boston Boston Massachusetts USA

**Keywords:** amphibian, critical periods, development, immune system, lymphoid organ, microbiome

## Abstract

Critical periods of development are time points that are especially sensitive to disruptions. Critical periods are important for microbiome assembly and the development of an effective immune system and, therefore, can have large and lasting impacts on host health, even through later life stages. Here, we investigated how a disruption to the microbiome of amphibians (treatment with a cocktail of six antibiotics and one antifungal compound) during early life and subsequent introduction to microbes at different developmental stages influences microbiome assembly and the development of the immune system (lymphoid tissues thymus and spleen). We found that antimicrobial treatments and introduction to microbes altered microbiome assembly (total microbial richness, antifungal microbial richness, composition, and relative abundances) and these changes were dependent on the timing of microbial introduction. Tadpoles treated with antimicrobials and then introduced to microbes at different developmental stages also had higher scaled abundances of bacteria in the phylum Actinobacteriota. However, after 7.5 weeks of tadpole development, we found no effects of treatment on lymphoid organ (thymus and spleen) size or on lymphoid cell counts. Overall, these results suggest that a disruption to the microbiome during early development, and more specifically, the length of the disruption and timing of reintroduction to microbes, can have significant impacts on microbiome assembly, potentially leading to long term impacts on host health.

## Graphical abstract

We used a novel amphibian gnotobiotic system to investigate how disruptions to the microbiome during critical developmental periods influence microbial assembly and the development of immunity. We found that antimicrobial treatments of embryos and subsequent introduction to microbes at different developmental stages altered microbiome assembly, including diversity, composition, and antifungal richness of the tadpole microbiome.

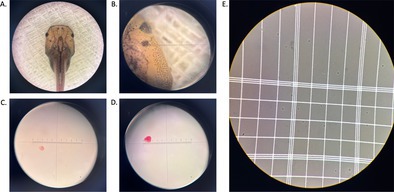

## 1 Introduction

Critical periods of development are time points early in the lives of organisms that are especially sensitive to disruptions (Selevan et al. 2000). Disruptions during these sensitive periods can cause lasting effects, some of which may even persist throughout adulthood (Selevan et al. 2000). Disruptions during critical periods have the potential to affect many different aspects of health, ranging from adult metabolism to cardiovascular function (Kuneš and Zicha 2006; Cox et al. 2014). These periods can be especially important for the development of immunity (Palmer 2011). For example, the weaning period can be a critical period for immune system development (Al Nabhani and Eberl 2020). Mice that experience a disruption to their microbiome during this period have increased susceptibility to disorders such as inflammatory bowel disease (IBD) in adulthood (Al Nabhani and Eberl 2020). As such, critical periods of development are an important factor in both immunological and microbiome research, as their potential impact on the immune system and overall host health can extend beyond the window of development (Al Nabhani and Eberl 2020).

The timing and order of exposure to microbes during critical periods are important for the development and structuring of the microbiome (Coyte et al. 2021). Different factors can influence microbiome assembly, such as the early life developmental environment (Campos‐Cerda and Bohannan 2020; Busi et al. 2021; McGrath‐Blaser et al. 2021; Jani and Briggs 2018). For example, in developing amphibians, microbiome composition and diversity are shaped both by host genetic background and by microbes present in the environment (McGrath‐Blaser et al. 2021; Jani and Briggs 2018). In humans, birth mode can also influence infant microbiome assembly, with birth by cesarean section known to alter microbiome composition and diversity (Busi et al. 2021). Microbiome assembly during development can have important implications for long‐term host health because alterations to microbiome composition and species abundances can influence the efficacy of the developing immune system (Al Nabhani and Eberl 2020; Gensollen et al. 2016).

Exposure to a diversity of microbes during critical periods is essential for the development of an effective immune system (Al Nabhani and Eberl 2020; Gensollen et al. 2016). Studies using germ‐free model systems, such as mice, have shown that individuals that develop without a microbiome during critical periods can have underdeveloped lymphoid tissues (e.g., Peyer's patches, mesenteric lymph nodes) and reduced lymphoid cell counts (Al Nabhani and Eberl 2020; Gensollen et al. 2016). Further, germ‐free mice have also been shown to have a higher prevalence of asthma compared to mice with functional microbiomes (Olszak et al. 2012; Gensollen et al. 2016; Stiemsma and Turvey 2017). This increased susceptibility to asthma is often irreversible, even with reintroduction to microbes later in life (Olszak et al. 2012; Gensollen et al. 2016; Stiemsma and Turvey 2017). Asthma prevalence in germ‐free mice therefore may only be reduced through colonization with microbes during critical neonatal periods (Olszak et al. 2012; Gensollen et al. 2016; Stiemsma and Turvey 2017). Thus, model mammalian germ‐free systems have been instrumental in showing that the microbiome is essential for the development of functional immunity, and the effects of the timing of exposure to microbes during critical developmental windows are complex and dynamic, with important research still emerging (Al Nabhani and Eberl 2020; Gensollen et al. 2016; Stiemsma and Turvey 2017).

Given the complex role microbial exposure plays during critical developmental periods and its importance in shaping immune function, it is beneficial to continue to develop novel model systems that allow for the precise manipulation of the microbiome during these windows (Gensollen et al. 2016; Stiemsma and Turvey 2017). Amphibians are emerging as non‐mammalian model organisms that can be useful for studying the importance of critical periods of development in the context of immune system maturation (Miller et al. 2023). This is because many amphibians have biphasic life histories, and development can be separated from the influence of parental care (Tobias et al. 1998). The decoupling of developing offspring from parents is important as it allows for the removal of the influence of the parental microbiome and investigation on the role of the offspring microbiome alone, a challenge with current model organisms (e.g., mice and pigs; Konig and Markl 1987, Andersen et al. 2011). Amphibians also have complex immune systems with both innate and adaptive branches (Grogan et al. 2018). Research on amphibian immunity is currently relevant in a conservation biology context as they are threatened by infectious diseases including chytridiomycosis caused by the fungal pathogen *Batrachochytrium dendrobatidis* (Grogan et al. 2018). Further, the amphibian genus *Xenopus* has been an important historical model used in studies centered on immunology and developmental biology (Carotenuto et al. 2023). Overall, previous work using primarily mammalian germ‐free systems have shown that the microbiome shapes the immune system during critical periods of development (Al Nabhani and Eberl 2020; Gensollen et al. 2016). Building from this work, amphibians offer unique characteristics that yield a strong potential to generate new knowledge we are unable to acquire from current model systems (Konig and Markl 1987; Andersen et al. 2011; Grogan et al. 2018; Carotenuto et al. 2023).

Previous studies using *Xenopus* species have already contributed to knowledge on amphibian immune system development (Rollins‐Smith 1998). Studies have shown that larval amphibians develop the thymus and the spleen in early life and generate lymphocytes in these organs that increase until metamorphosis (Rollins‐Smith 1998). Previous studies using *Xenopus* have also suggested that the microbiome may be an important influence on amphibian development (Bishop and Beck 2021). For example, the amphibian skin microbiome has been shown to influence *Xenopus* tail regeneration during development (Bishop and Beck 2021). Previous studies using non‐*Xenopus* amphibians (e.g., *Osteopilus septentrionalis, Lithobates pipiens*) have also made important contributions to amphibian developmental research, suggesting that a disruption to the larval microbiome through the use of antimicrobials can affect development (i.e., organ growth; Emerson and Woodley 2024) and may also result in changes that persist after metamorphosis, such as increased susceptibility to disease (Knutie et al. 2017).

Previous studies have shown a connection between the microbiome and amphibian development, and that the microbiome shapes immunity in other model systems, however, to date, little is known about how the microbiome drives immune development in amphibians (Al Nabhani and Eberl 2020; Gensollen et al. 2016; Bishop and Beck 2021; Knutie et al. 2017). Therefore, the goal of this study was to examine how exposure to a diversity of microbes at varying critical developmental stages may affect amphibian immune development. We first reduced the microbiome of *Silurana* (*Xenopus*) *tropicalis* (hereafter, *X. tropicalis* ) embryos using a mixture of antimicrobials (Miller et al. 2023). We then reared tadpoles in sterile conditions for different lengths of time before removing them from sterile conditions, thereby exposing them to a greater diversity of microbial communities. We selected time points for microbial introduction that corresponded with development stages important for thymus and spleen development (Du Pasquier et al. 2000; Robert and Ohta 2009; Lee et al. 2013). We predicted that a disruption to the microbiome during early life and subsequent removal from sterile conditions at different life stages would (1) influence microbiome assembly during development (i.e., alter microbiome composition and diversity) and (2) alter lymphoid organ development (similar to changes previously observed in mice; Al Nabhani and Eberl 2020, Gensollen et al. 2016).

## 2 Materials and Methods

### 2.1 Embryo Transport

We received *X. tropicalis* embryos from the Marine Biological Laboratory, National Xenopus Resource (Woods Hole, MA, USA; Miller et al. 2023). The embryos arrived at approximately Nieuwkoop and Faber stage 16 (NF stage; approximately Gosner stage 14; Nieuwkoop and Faber 1994, Zahn et al. 2022). NF stages describe Xenopus development, similar to Gosner staging, however, NF stages are more specific and a better fit for Xenopus developmental studies (Nieuwkoop and Faber 1994; Zahn et al. 2022). Upon arrival, we randomly separated the embryos into two treatment groups and placed them into 50 mL conical tubes (approximately 60 embryos per conical tube). We moved one group inside a laminar flow biosafety 2 cabinet and kept the other outside the biosafety cabinet (i.e., in nonsterile conditions). We rinsed each group three times with sterilized (i.e., autoclaved) deionized (DI) water and then placed the group inside of the biosafety cabinet into an antimicrobial (AMX) cocktail (described below) to soak for 4.5 h (Miller et al. 2023). We treated the nonsterile control group outside the biosafety cabinet in the same manner but replaced the AMX cocktail with an equal volume of autoclaved water (Miller et al. 2023). The temperature during development was approximately 22°C, and we previously confirmed the temperature inside the biosafety cabinet did not differ from the temperature outside the biosafety cabinet (Miller et al. 2023).

### 2.2 Animal Husbandry

We allowed the tadpoles in each group to develop in petri dishes for approximately 2 weeks and then moved them to three stainless steel tanks per treatment group that were either autoclaved (for the sterile treatment groups) or not (approximate starting density of 45–50 tadpoles per 16.5 × 16 × 26.7 cm tanks; Thunder Group, CA, USA).

We fed the tadpoles *Xenopus* larvae tadpole powder (Carolina Biological; NC, USA) *ad libitum*. We sterilized the tadpole powder for the animals in the sterile treatment groups via gamma irradiation with a dose of 15 kGy, which we previously validated for sterility (Miller et al. 2023). We also spot cleaned the petri dishes and tanks as needed with sterile transfer pipettes and added fresh sterile or nonsterile water at least once per week. To prepare the tadpole water, we allowed DI water to dechlorinate for at least 24 h prior to use and then added an aquarium salt mixture (Instant Ocean; Blacksburg, VA, USA) until the conductivity of the water was approximately 1200 microsiemens. We then autoclaved the water for the sterile groups in groups of 1 L glass media bottles (Corning Life Sciences Pyrex; USA) and allowed it to cool to room temperature before adding it to the tanks.

### 2.3 Antimicrobial Treatments

The antimicrobial (AMX) cocktail consisted of 500 μL of penicillin G:streptomycin (10,000 units mL^−1^: 10 mg mL^−1^; antibiotic), 50 μL of amphotericin B solution (250 μg mL^−1^; antifungal), 200 μL kanamycin sulfate (25 ug mL^−1^; antibiotic), 0.53 μL of sulfamethoxazole: trimethoprim (13.3 mg L^−1^: 2.67 mg L^−1^; antibiotic), and 1.2 mg enrofloxacin (final concentration 30 mg L^−1^; antibiotic) added to an autoclaved 250 mL glass beaker with autoclaved DI water to create a total volume of 40 mL (Miller et al. 2023). We then homogenized and filter‐sterilized the AMX cocktail inside a biosafety cabinet using a 0.22 μm sterile syringe filter. Following the treatments with either AMX or the sham solution, we rinsed the embryos three additional times with sterile water. We then moved the embryos to their respective sterile petri dishes either inside or outside the biosafety cabinet with 50 mL of either sterile or nonsterile water with aquarium salt, depending on treatment group.

After the AMX and sham treatments, we randomly and evenly separated the embryos (approximately *N* = 220–240 embryos per treatment group) inside the biosafety cabinet into three additional groups. We used a labeling system, denoted by “AMX_No.”, for each group that reflected if the group received AMX or sham treatments and the length of time in sterile conditions (No. of days). We held the first group inside the biosafety cabinet and in sterile conditions (i.e., fed sterile food, held in sterile water) from embryo arrival to the lab throughout the duration of the entire experiment (i.e., through week 10 of the experiment; AMX). We held a second group inside the biosafety cabinet and in sterile conditions from embryo arrival to the lab until initial thymus development, or, approximately experimental day 5 (Du Pasquier et al. 2000; Robert and Ohta 2009; Lee et al. 2013), when we then removed the tadpole tanks from inside the biosafety cabinet, fed the group nonsterile food, and maintained them in nonsterile water (AMX_5). This change in husbandry exposed them to the various microbes present in the nonsterile environment. We maintained the next group inside the biosafety cabinet in sterility from embryo arrival to the lab until experimental day 25, when we then removed the tadpole tanks from inside the biosafety cabinet and exposed them to nonsterile conditions (AMX_25). This time point of removal from sterile conditions corresponded with the development of both the thymus and spleen (Du Pasquier et al. 2000; Robert and Ohta 2009; Lee et al. 2013). We also included a nonsterile, conventionally raised treatment group that was not treated with antimicrobials and held in nonsterile conditions for the entirety of the experiment (i.e., fed nonsterile food and maintained in nonsterile water; No AMX). A visual schematic of the experimental design is included in the Supporting Information (Figure S1).

### 2.4 Tadpole Microbiome DNA Extractions

Five weeks after initial embryo arrival to the lab, we haphazardly selected *N =* 10 tadpoles per treatment group (approximately NF stage 55–56; Nieuwkoop and Faber 1994) for whole‐body 16S rRNA targeted amplicon microbiome sequencing analysis (Miller et al. 2023). We first filter‐sterilized MS‐222 and humanely euthanized the tadpoles (National Research Council. Committee on Care, and Use of Laboratory Animals 1986). We measured tadpole mass to the nearest 0.1 g and snout‐vent length (hereafter, SVL) to the nearest 0.1 mm. We homogenized the tadpoles using the Disruptor Genie homogenizer (Scientific Industries, Bohemia, NY, USA) and used the QIAmp PowerFecal Pro DNA Kit (Qiagen, Valencia, CA, USA) to extract DNA. We homogenized the tadpoles and extracted the DNA following the Qiagen protocol associated with the QIAmp PowerFecal Pro DNA Kit.

### 2.5 Sequencing and Sequence Data Pre‐Processing

We sent extracted DNA to the Idaho State University Molecular Research Core Facility (RRID:SCR_012598) for sequencing and included *N* = 4 water blanks (i.e., molecular grade water extracted using the same QIAmp DNA Kit). For sequencing, a bacterial 16S rRNA gene fragment from the V4 region was polymerase chain reaction (PCR) amplified from each DNA extract and was sequenced on the Illumina MiSeq (Illumina Inc., San Diego, California, USA) using methods previously described (Miller et al. 2023). For all microbiome data apart from the antifungal richness analysis (described below), demultiplexed FASTQ files, with primers and adapters removed, were processed in R (v. 4.4.1; R Core Team, n.d.) using the dada2 pipeline (v. 1.32.0; R Core Team, n.d.). Reads were trimmed, merged, and chimeras removed. We performed taxonomic assignment using the SILVA bacterial 16S rRNA database (v. 138.1; Quast et al. 2012).

### 2.6 Tadpole Dissections

We haphazardly selected *N* = 10 tadpoles from all treatment groups approximately 7.5 weeks after initial embryo arrival in the lab. We chose this time point because the tadpoles were large enough to dissect while not yet actively going through metamorphosis, when their lymphocyte numbers are known to decrease (Rollins‐Smith 1998). We euthanized the tadpoles using MS‐222 and measured mass and SVL (Miller et al. 2023). We then measured the size of both the left and right thymus using a dissecting microscope and an eyepiece with a reticle at 30× total magnification (AmScope 10× Microscope Eyepiece with Reticle 23 mm; AmScope, CA, USA). We dissected both the thymuses and added them to 200 μL of sterile amphibian phosphate‐buffered saline (APBS; 25 mL H_2_O, 100 mL PBS; Edholm 2018). We homogenized the thymuses in the liquid by gently pulling them apart using fine‐tipped forceps and counted the thymus cells present in the solution using a hemocytometer (Edholm 2018; Rollins‐Smith et al. 1996). We dissected the spleens using fine‐tipped forceps and micro‐dissection scissors, floated them in 200 μL of APBS, and measured the diameter using a dissecting microscope and an eyepiece with a reticle at 30× total magnification (Edholm 2018; Rollins‐Smith et al. 1996). To calculate the diameter of both the thymuses and spleens, we converted the eyepiece reticle measurements to micrometers using a microscope stage calibrator (AmScope Microscope Stage Calibration Slide; AmScope, CA, USA).

### 2.7 Statistical Analyses

We conducted statistical analyses using R (v. 4.0.2; R Core Team, n.d.), choosing to run nonparametric tests when the assumption of normality was violated. We ran a nonparametric Kruskal–Wallis test with a *post hoc* Dunn test to look for differences in two alpha diversity metrics (Chao1 and Shannon diversity). We used the Chao1 metric as a nonparametric measure of species richness and Shannon diversity as a measure of both richness and evenness (Kim et al. 2017). We assessed beta diversity by calculating the Bray–Curtis dissimilarity to quantify compositional differences between samples. We performed non‐metric multidimensional scaling (NMDS) ordination using the phyloseq (v. 1.48.0) package. We generated ordinations from Bray–Curtis dissimilarity and Jaccard distance calculated from relative abundance (proportional) data, and experimental groups were visualized using 95% confidence ellipses. We tested community composition differences among groups using permutational multivariate analysis of variance (PERMANOVA; 999 permutations) and assessed differences in multivariate dispersion using permutational analysis of multivariate dispersion (PERMDISP), with *post hoc* Tukey's HSD tests. We used the vegan package (v. 2.7) to conduct all multivariate analyses. We evaluated differential taxonomic abundance at the amplicon sequence variant (ASV) level using Analysis of Compositions of Microbiomes with Bias Correction (ANCOM‐BC2), in the ANCOMBC package (v. 2.12.0), with Holm‐adjusted *p* values used to control for multiple testing across taxa. The phylum Actinobacteriota was differentially abundant in the AMX_25 group, and given its relevance to our research questions, we further examined this phylum using targeted group comparisons. We analyzed relative abundance data using a Kruskal–Wallis test, followed by pairwise Wilcoxon rank sum test with Holm adjusted *p* values.

We used the AmphiBac v. 2023.2 database to determine the antifungal richness and the percent of antifungal bacteria present in the microbiome (Bletz and Woodhams 2023; expanded from Woodhams et al. 2015). We chose to look at this metric as a proxy for immune function because the richness of bacteria in the microbiome with antifungal capabilities is a known indicator of susceptibility to disease in amphibians (Woodhams et al. 2023; Nava‐González et al. 2021; Jiménez et al. 2022). We first generated representative sequences from samples using the QIIME 2 platform paired‐end sequence pipeline, trimming sequences at 250 bp based on quality scores as well as previous studies (Estaki et al. 2020; Schuck et al. 2024). We then matched our bacterial sequences using a 99% similarity to the AmphiBac database of skin bacteria previously tested in culture for the ability to inhibit the growth of a fungal pathogen (i.e., *Batrachochytrium dendrobatidis*; Woodhams et al.^2015^). We rarified the data to the minimum sequencing depth and calculated antifungal bacterial richness (i.e., the richness of bacteria that matched to bacteria in the AmphiBac database) and the percent of antifungal bacteria present in the microbiome (i.e., the number of antifungal bacterial sequences divided by the total number of sequences for each sample, or, the proportion of antifungal bacteria). We then compared the percent of predicted antifungal bacteria and antifungal bacterial richness among each treatment group with a non‐parametric Kruskal–Wallis test and *post hoc* Dunn tests using Holm adjusted *p* values.

For all body condition data, we first calculated body condition as tadpole mass divided by tadpole SVL and then ran ANOVAs with *post hoc* Tukey's HSD tests to look for pairwise differences among treatment groups. For the thymus diameter data, we took the average diameter of the two thymuses for each tadpole. Then, for the thymus diameter, spleen diameter, and thymus cell count data, we ran non‐parametric Kruskal–Wallis tests with *post hoc* Dunn tests to look for pairwise differences using Holm adjusted *p* values. Lastly, we plotted the diameter of the thymus (*y*‐axis) against tadpole mass (*x*‐axis) to see if there was a relationship between tadpole size and thymus size. We ran a Pearson's correlation, a linear model including a mass by treatment group interaction, and then a *post* hoc ANOVA to look for pairwise differences among treatment groups.

## 3 Results

### 3.1 Microbiome Alpha Diversity

**FIGURE 1:**
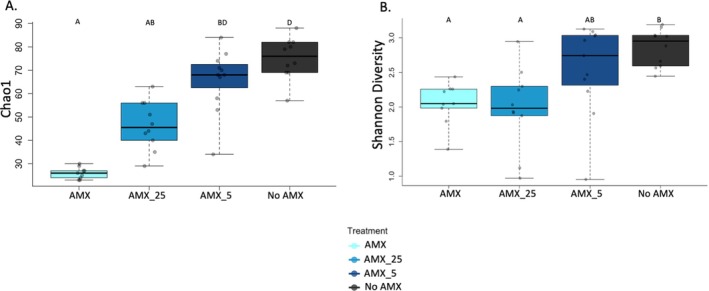
Box plots showing (A) Chao1 index, a measure of species richness, and (B) Shannon diversity index, which accounts for both richness and evenness. Each plot displays the median with upper and lower quartiles and maximum and minimum values for each experimental condition, ordered from most to least sterile exposure (*n* = 10).

We found differences in alpha diversity among our treatment groups. Using the Chao1 index, we found a gradient of decreased microbial richness with our antimicrobial treated groups, with the AMX group having lowest richness and the nonsterile group (No AMX) having the highest microbial richness (Figure 1A; Kruskal–Wallis: $\chi_{3}^{2}$ = 30.54, *p* < 0.05; AMX and AMX_5 Dunn test *p* < 0.05; AMX and No AMX Dunn test *p* < 0.05; AMX_25 and No AMX Dunn test *p* < 0.05). We found no significant differences in Chao1 indices between the AMX group and AMX_25 or between the AMX_25 and AMX_5 groups (AMX and AMX_25 Dunn test *p* = 0.10; AMX_25 AMX_5 Dunn test *p* = 0.11). We also found no significant differences in Chao1 indices between AMX_5 and the nonsterile group (AMX_5 and No AMX Dunn test *p* = 0.24).

We also found differences in Shannon diversity among our treatment groups. Specifically, we found the AMX group and the AMX_25 group had significantly lower Shannon diversity compared to the nonsterile treatment group (Figure 1B; Kruskal–Wallis: 𝜒^2^_3_ = 17.46, *p* < 0.05; AMX and No AMX Dunn test *p* < 0.05; AMX_25 and No AMX Dunn test *p* < 0.05). However, we found no significant differences in Shannon diversity among any of the antimicrobial treated groups or between the antimicrobial group removed from sterile conditions on day 5 (AMX_5) and the nonsterile group (AMX and AMX_25 Dunn test *p* > 0.05; AMX and AMX_5 Dunn test *p* = 0.07; AMX_25 and AMX_5 Dunn test *p* = 0.09; AMX_5 and No AMX Dunn test *p* > 0.05).

### 3.2 Microbiome Community Composition

**FIGURE 2:**
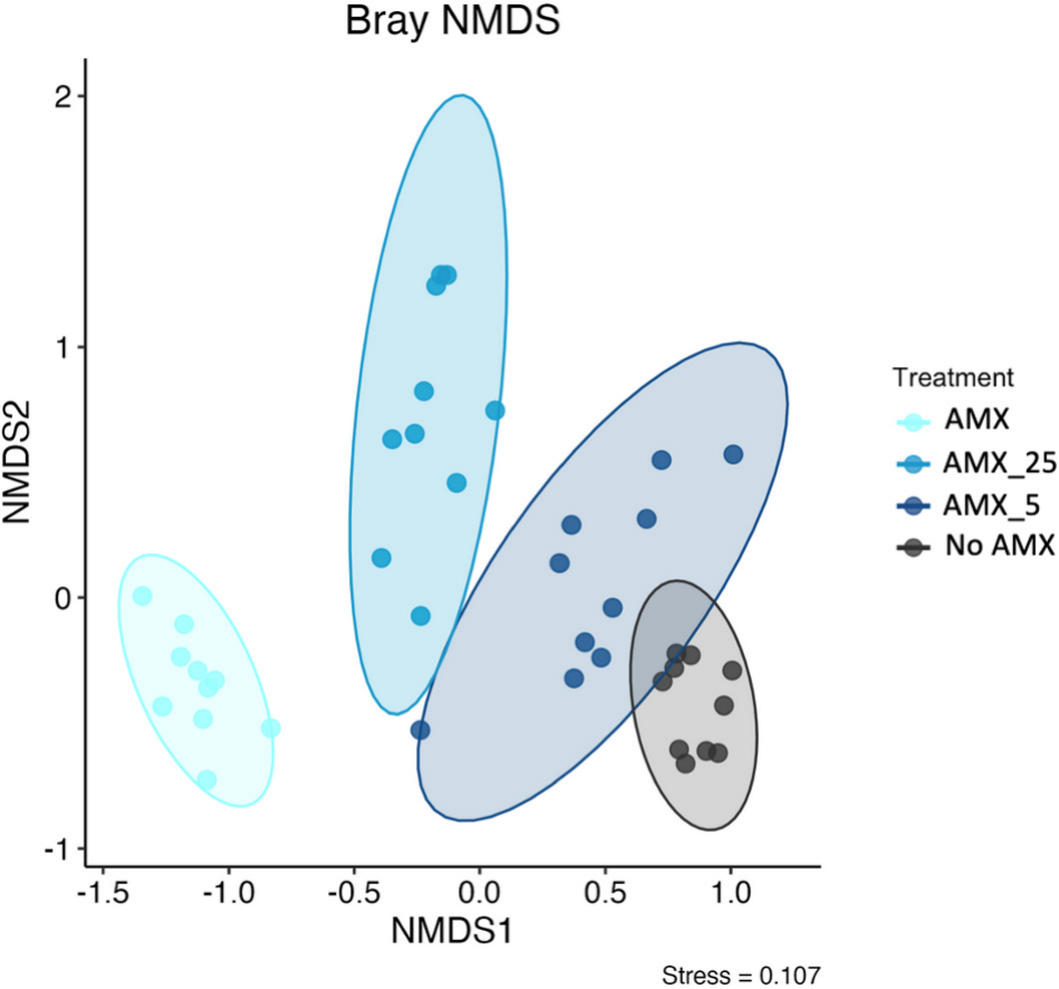
Bray‐Curtis NMDS plot for *Xenopus tropicalis* treated with either an antimicrobial cocktail or a sham control solution and removed from sterile conditions at various time points during development. DNA was extracted from tadpoles 5 weeks after the embryos initially arrived in the laboratory.

Experimental groups differed significantly in microbiome composition (PERMANOVA: Bray‐Curtis: *R* ^2^ = 0.62, *p* < 0.05; Jaccard: *R* ^2^ = 0.51, *p* < 0.05). Visualization using the NMDS plots revealed our groups formed different clusters (Figure 2; Figure S2; stress = 0.107); the antimicrobial group continuously maintained in sterile conditions and the antimicrobial group removed from sterile conditions on day 25 formed distinct clusters from the other treatment groups. In addition, the antimicrobial group removed from sterile conditions on day 5 and the nonsterile group were positioned closer together with community overlap. When examining relative abundances, we saw treatment groups did not differ in microbiome dispersion (PERMDISP: Bray‐Curtis F_3,36_ = 1.58, *p* = 0.196), indicating similar within‐group variability. However, when examining bacterial membership (presence/absence), the group continuously maintained in sterile conditions (AMX) showed significantly lower dispersion than all other groups (PERMDISP: Jaccard F_3,36_ = 12.28, *p* < 0.05; Tukey's HSD all pairwise *p* < 0.02; Table S2).

**FIGURE 3:**
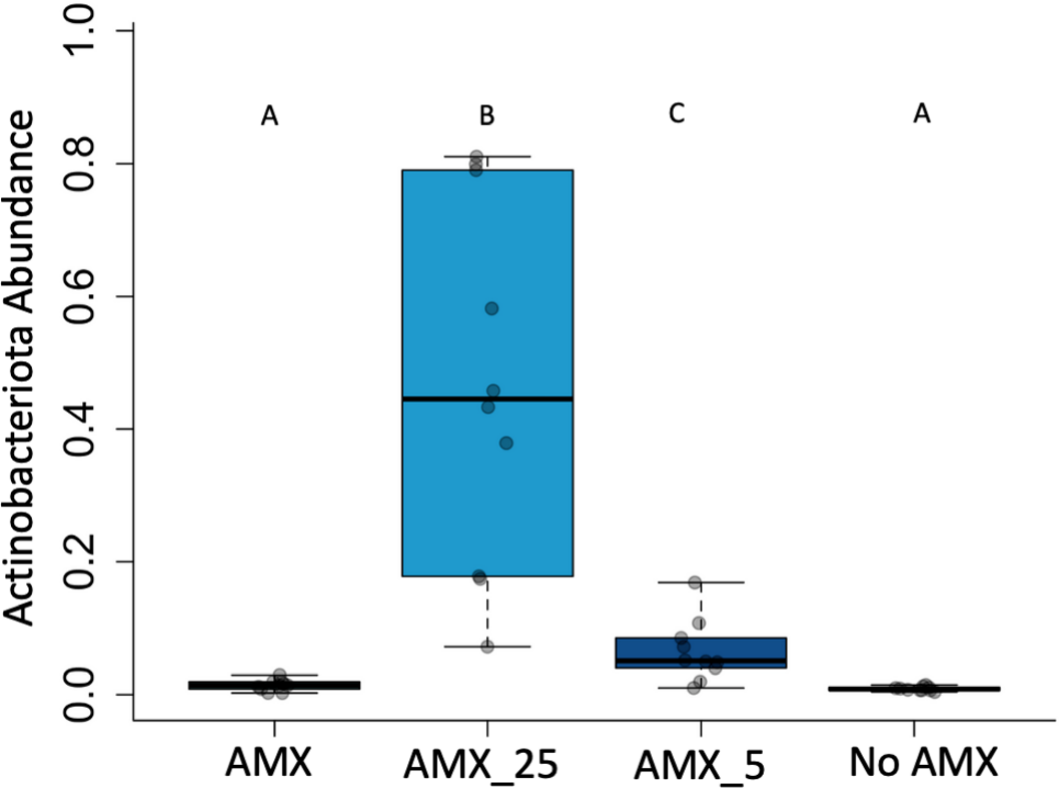
Scaled abundances of phylum Actinobacteriota in groups of *Xenopus tropicalis* treated with either an antimicrobial cocktail or a sham control solution and removed from sterile conditions at various time points during development. DNA was extracted from tadpoles 5 weeks after the embryos initially arrived in the laboratory.

Differential abundance analysis (ANCOM‐BC2) identified significantly different taxa across pairwise comparisons (Table S1). At the phylum level, Actinobacteriota was significantly more abundant the groups removed from sterile conditions on day 25 and day 5 relative to the nonsterile group (AMX_25: Log Fold Change = 4.59, *q* < 0.001; AMX_5: Log Fold Change = 1.79, *q* < 0.001). Specifically, we found the highest scaled abundance of Actinobacteriota in the antimicrobial group removed from sterile conditions on day 25 (Figure 3; Figure S3; Kruskal–Wallis: $\chi_{3}^{2}$ = 30.98, *p <* 0.05; Wilcoxon rank sum test for AMX_25 compared to all other groups *p* < 0.05). We saw the second highest abundance in the antimicrobial group removed from sterile conditions on day 5 (Wilcoxon rank sum test for AMX_5 compared to all other groups *p* < 0.05). We saw no differences in the scaled abundance of the phylum Actinobacteriota between the antimicrobial group continuously maintained in sterile conditions and the nonsterile group (Wilcoxon rank sum test AMX and No AMX *p* > 0.05).

### 3.3 Predicted Microbiome Antifungal Function

We found a significantly higher percent of antifungal bacteria in the group removed from sterile conditions on day 5 compared to the group removed on day 25, and this was the only significant difference in the percent of antifungal bacteria among the groups (Figure S4; Kruskal–Wallis: $\chi_{3}^{2}$ = 9.59, *p* = 0.02; AMX_25 and AMX_5 Dunn test *p* = 0.02).

We also found differences in the richness of antifungal bacteria of the tadpole microbiome, in a pattern that mirrored our overall microbial richness results (Figure S4; Kruskal–Wallis: $\chi_{3}^{2}$ = 31.28, *p* < 0.05). We found a significantly lower antifungal bacteria richness in the AMX group compared to both the AMX_5 group and the nonsterile group (AMX and AMX_5 Dunn test *p* < 0.05; AMX and No AMX Dunn test *p* < 0.05). We also found the antimicrobial group removed from sterile conditions on day 25 had a lower antifungal richness than the nonsterile treatment group (AMX_25 and No AMX Dunn test *p* < 0.05).

### 3.4 Body Condition

We compared body condition among our treatment groups for three different time points during development (i.e., week 5 of development, week 7 of development, and week 11 of development). We found no differences in body condition among our treatment groups during any time point (Figure S5; ANOVA: *F*_3,36_ = 0.57, *p* > 0.05; ANOVA: *F*_3,36_ = 3.03, *p* = 0.04, Tukey HSD all groups *p* > 0.05; ANOVA: *F*_3,36_ = 0.72, *p* > 0.05).

### 3.5 Lymphoid Organ Development

**FIGURE 4:**
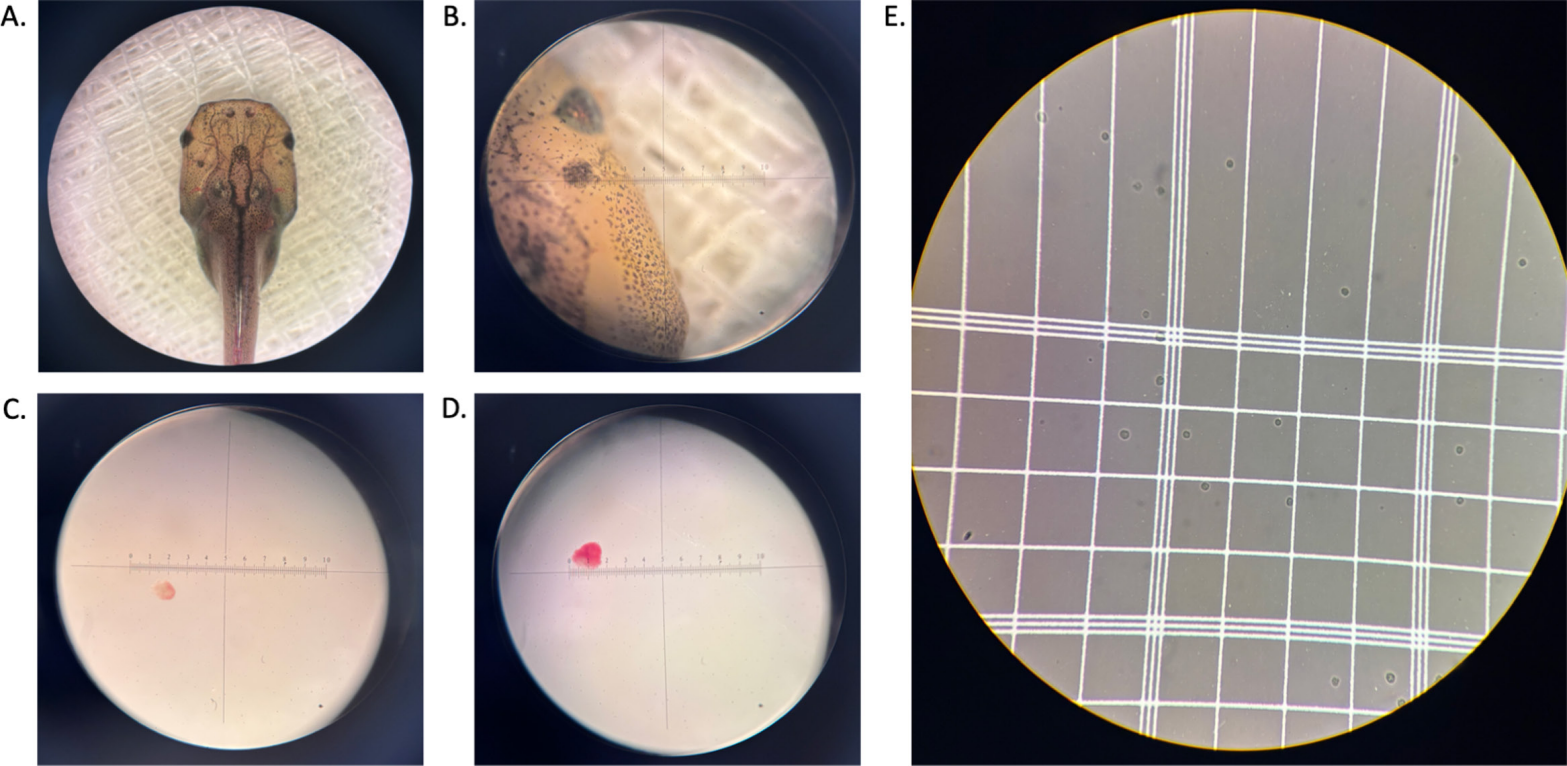
Pictures of *Xenopus tropicalis* tadpoles during dissections 7.5 weeks after the embryos initially arrived in the laboratory. (A) Picture of *X. tropicalis* tadpole prior to dissection. (B) Picture of *X. tropicalis* thymus prior to dissection. Taken with an eyepiece with a reticle at 30× total magnification. (C, D) Picture of *X. tropicalis* spleens after dissection. Taken with an eyepiece with a reticle at 30× total magnification. (E) Picture of *X. tropicalis* thymocytes in a hemocytometer.

**FIGURE 5:**
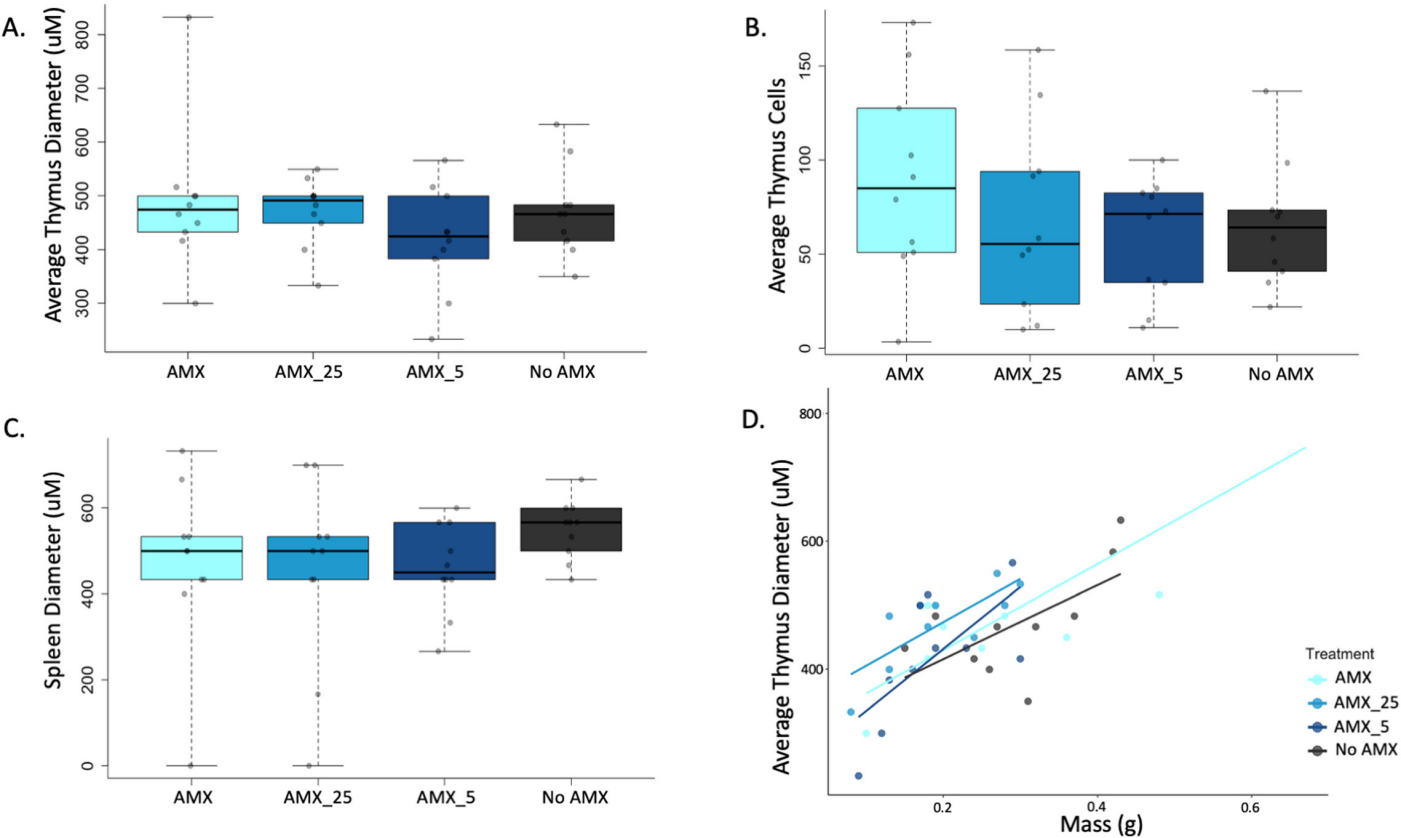
Average thymus diameter, average thymocyte cell counts, spleen diameter, and average thymus diameter by tadpole mass taken 7.5 weeks after the embryos initially arrived in the laboratory. (A) Thymus diameter for *X. tropicalis* tadpoles taken as the average diameter of the two thymuses for each tadpole. (B) Average thymocyte cell counts for cells in both thymuses for each tadpole, counted using a hemocytometer. (C) Spleen diameter for *X. tropicalis* tadpoles. Spleens that were too small to measure were denoted 0. (D) Average thymus diameter by mass of *X. tropicalis* tadpoles.

We compared the average diameter of the thymus, the diameter of the spleen, and the number of thymocytes among our treatment groups (Figure 4). We found no differences in thymus diameters (Figure 5A; Kruskal–Wallis: $\chi_{3}^{2}$ = 2.53, *p* > 0.05), in spleen diameters (Figure 5B; Kruskal–Wallis: $\chi_{3}^{2}$ = 3.83, *p* > 0.05), or in thymocyte numbers (Figure 5C; Kruskal–Wallis: $\chi_{3}^{2}$ = 1.79, *p* > 0.05) among treatment groups.

We found a significant relationship between tadpole mass and thymus diameter (Figure 5D; Pearson's correlation: *r* = 0.74, *p* < 0.05; ANOVA: *F*_1,32_ = 46.72, *p* < 0.05). However, we found no significant effect of treatment (ANOVA: *F*_3,32_ = 1.66, *p* > 0.05) or of a mass by treatment group interaction (ANOVA: *F*_3,32_ = 0.32, *p* > 0.05).

## 4 Discussion

Microbiome assembly in early life stages is thought to be important in influencing organismal health (Campos‐Cerda and Bohannan 2020; Busi et al. 2021). This is likely because exposure to a diversity of microbes during early developmental windows plays a key role in the maturation of an effective immune system (Al Nabhani and Eberl 2020; Gensollen et al. 2016). Disruptions to the microbiome during these critical developmental periods can have impacts that last throughout an individual's entire life (Stiemsma and Turvey 2017). Here, we investigated both the effect of a disruption to the microbiome and the timing of reintroduction to microbes on microbiome assembly and the development of immunity using an amphibian model system (Miller et al. 2023).

Our results indicate that the timing and duration of exposure to microbes during development are key factors shaping tadpole microbiome assembly. Tadpoles exposed to microbes later in development (AMX_25) had a microbiome diversity more similar to that of the antimicrobial group continuously maintained in sterile conditions (AMX). Tadpoles exposed to microbes early in development (AMX_5) had a microbiome diversity more similar (and, with community overlap) to that of the nonsterile, conventionally raised treatment group (No AMX). These results suggest that the microbiome was still under assembly during the earlier developmental stages and was therefore more able to recover from a disruption (i.e., antimicrobial treatment). This finding also suggests that the microbiome was less able to recover from disruptions later in life, after this period of early life microbial structuring.

Rather than one distinct time point that is most important for microbiome assembly, these results suggest that there may be a gradient of vulnerability to disruptions such that sensitivity shifts over the course of development, an idea we refer to as the Gradient of Developmental Vulnerability Hypothesis. Accordingly, individuals may also be better able to recover from disruptions that are shorter in duration than they would from longer duration disruptions during development. Previous studies have often identified a single, discrete critical period during development where disruptions before or after this period can lead to irreversible changes to the host, such as higher susceptibility to disease in later life (e.g., the weaning period; Al Nabhani and Eberl 2020, Stiemsma and Turvey 2017, Knutie et al. 2017). Our results build off this body of research, further suggesting that microbiome disruption may not always yield a strictly dichotomous result (i.e., disruptions before this period lead to changes in the host and after this period do not). Further refinement of the Gradient of Developmental Vulnerability Hypothesis would require comparing tadpole microbiome disruption over additional time points.

In addition to changes to the diversity of the microbiome, the timing and duration of microbial exposure can also influence the composition of the tadpole microbiome. Our treatment groups had differential scaled abundances of the phylum Actinobacteriota present in their microbiomes, with the treatment groups removed from sterile conditions on day 5 and day 25 of the experiment having significantly higher levels of these bacteria. Moreover, these two groups had higher abundances of the Nocardiaceae family (p: Actinobacteria; f: Nocardiaceae), with the group removed from sterile conditions later in development having higher levels than all other treatment groups. This suggests that the disruption to the microbiome may have led to microbial dysbiosis, with the largest effect in the group that experienced the longest disruption to the microbiome, providing further support for the Gradient of Developmental Vulnerability Hypothesis. The subsequent re‐exposure to microbes may have allowed for more successful establishment of Nocardiaceae bacteria. We do not expect this was a result of contamination as the Nocardiaceae family was present in all groups at relatively low abundances and peaked in the treatment groups removed from sterile conditions on day 5 and day 25.

Nocardiaceae bacteria can be opportunistically pathogenic, with bacteria in the genus Nocardia (p: Actinobacteria; f: Nocardiaceae, g: Nocardia) causing the disease nocardiosis in a wide range of host species (e.g., humans, cows, dogs, cats, horses, birds (Mehta and Shamoo 2020; Churgin et al. 2019; Ribeiro et al. 2008; Malik et al. 2006; Biberstein et al. 1985)) under specific conditions (Mehta and Shamoo 2020; Churgin et al. 2019). One major contributing factor influencing susceptibility to nocardiosis is altered immunity (Mehta and Shamoo 2020). This makes nocardiosis relevant in a microbiome context, as disruptions to the microbiome can alter immune function, which could allow for more successful establishment of opportunistic pathogens (Al Nabhani and Eberl 2020; Gensollen et al. 2016). We primarily identified the change in Nocardiaceae bacteria in our treatment groups as the Rhodococcus genus (p: Actinobacteria; f: Nocardiaceae, g: Rhodococcus), however, this example highlights that developmental disruption‐induced changes to the composition of the microbiome may affect host health by leading to microbial dysbiosis, potentially causing increased disease susceptibility, altered immunity, or opportunistic infection (Al Nabhani and Eberl 2020; Gensollen et al. 2016; Mehta and Shamoo 2020; Churgin et al. 2019).

Previous work has also connected an early life disruption to the microbiome to microbial dysbiosis and altered host health (Miller et al. 2023; Knutie et al. 2017; Warne et al. 2019; Lynn et al. 2018). Antibiotic treatments administered to developing mice have been shown to lead to dysbiosis, ultimately leading to impaired antibody responses in later life (Lynn et al. 2018). In amphibians, early life treatment with antibiotics has been connected to changes in the microbiome and differences in growth, survival, developmental time, and susceptibility to disease in some cases (Knutie et al. 2017; Warne et al. 2019). However, in other cases, an early life disruption to the microbiome has not led to increased disease susceptibility in amphibians, highlighting the complexity of this topic (Miller et al. 2023). Together, this work underscores the important potential effects of microbial dysbiosis on host health and indicates that more work is needed in the amphibian microbiome system to fully elucidate species‐specific and context dependent effects (Miller et al. 2023; Knutie et al. 2017; Warne et al. 2019; Miller and Voyles 2026).

In addition to microbial dysbiosis, early life antimicrobial treatment can change the richness of the predicted antifungal bacteria in the microbiome (Miller and Voyles 2026). In amphibians, the richness of antifungal bacteria in the microbiome is a known influence on susceptibility to chytridiomycosis (Nava‐González et al. 2021; Jiménez et al. 2022; Miller and Voyles 2026). In fact, many studies have shown a correlation between decreased antifungal bacterial richness, higher infection prevalence, and higher pathogen loads in infected individuals (Nava‐González et al. 2021; Jiménez et al. 2022). In addition to a decrease in the overall richness of the tadpole microbiome, our results also showed a decrease in the richness of the antifungal bacteria, with the magnitude of this effect dependent on the timing of introduction to microbes. This could be significant for amphibians currently threatened by fungal pathogens such as *Batrachochytrium dendrobatidis*, because of the well characterized impact of the antifungal bacteria in the amphibian microbiome on susceptibility to chytridiomycosis (Nava‐González et al. 2021; Jiménez et al. 2022; Miller and Voyles 2026). Additional features of microbial communities that may differ among treatments that would be informative to study include network structure and centrality (Rawstern et al. 2025). Another additional future point for exploration would be to determine if these changes to the antifungal potential of the microbiome that occurred during development persist through metamorphosis, which may have broader implications for amphibian population health and susceptibility to disease.

Although our treatments were successful at manipulating microbiome assembly during sensitive developmental periods, we did not detect differences in lymphoid organ size or lymphocyte cell counts that would have provided additional support for alterations in immune system development and/or immune impairment. This indicates that changes to the microbiome in early life may not result in changes to all aspects of developing immunity, highlighting the need for more research to understand the full impact of microbiome‐host interactions. We chose to look at tissues involved in adaptive immunity because of the microbiome's known role in its development in other systems (Al Nabhani and Eberl 2020; Gensollen et al. 2016). However, early life disruptions to the microbiome may still affect aspects of amphibian immunity untested in this study (Grogan et al. 2018; Myers et al. 2012). For example, it is known that commensal bacteria in the amphibian microbiome can bolster the inhibitory effectiveness of the antimicrobial peptides (AMPs) present in skin secretions that are part of the innate immune system (Myers et al. 2012). Because bacteria are known to play an important role in increasing the effectiveness of AMPs, future studies addressing how skin secretions are affected by an early life disruption to the microbiome could yield interesting results (Myers et al. 2012).

This study underscores that amphibians are a compelling and unique model organism for further work uncovering the role of the microbiome in immune system development (Miller et al. 2023; Miller and Voyles 2026). We expect that amphibians, with their biphasic life histories, will make important contributions to this field of study because of the ability to easily manipulate the microbiome, the accessibility of lymphoid organs (i.e., the ability to measure the thymus through the skin), and the capacity for rearing tadpoles with the absence of parental care (Miller et al. 2023; Tobias et al. 1998; Konig and Markl 1987; Andersen et al. 2011; Rollins‐Smith et al. 1996). Here, we have optimized a system to examine critical periods in tadpole development, where exposure to diverse microbes early enough in development may allow for successful microbiome assembly, and exposure to microbes after this period may affect microbiome assembly in ways that could persist throughout development. We also build off the classical idea of critical periods of development by positing the idea of a gradient of developmental vulnerability to explain the results from this project, where sensitivity to disruptions during development changes over time and does not “start” or “stop” at discrete intervals, and where the duration of a disruption affects the magnitude of effect (i.e., a longer disruption leads to a more affected microbiome).

By optimizing a system that allows for manipulation of the amphibian microbiome during critical periods of development, we can begin to understand microbial assembly and the microbiome's effect on immunity in amphibians. Future work is needed to investigate if these effects on microbiome assembly persist through metamorphosis, as the amphibian microbiome is known to be restructured during metamorphosis (Kohl et al. 2013), and if the disruptions to the microbiome during early life may affect untested aspects of immunity (Myers et al. 2012). Lastly, it would be interesting to test additional methods of sterilization (Vismara et al. 2006; Huyck et al. 2015; Fainsod and Kot‐Leibovich 2018; Retnaningdyah and Ebert 2016). For example, sterilizing agents including rinses in ethanol, hydrogen peroxide, or mercury compounds are potential alternatives to antibiotic reduction of microbiota from *Xenopus* embryos but may have developmental consequences (Vismara et al. 2006; Huyck et al. 2015; Fainsod and Kot‐Leibovich 2018). For organisms such as *C. elegens* and *Daphnia*, bleach solutions can be effective at sterilizing eggs, but these eggs are typically better protected than amphibian embryos (Retnaningdyah and Ebert 2016; Rendueles et al. 2012). Thus, future studies could investigate the efficacy of dejellying with LCysteine hydrochloride (Wlizla et al. 2018), as an antimicrobial alternative or in addition to antibiotics. Ultimately, the diversity and composition of the microbiome are key in priming the developing vertebrate immune system (Al Nabhani and Eberl 2020; Gensollen et al. 2016). Because of this, the generation of information to better understand how the developmental environment can affect microbial assembly is essential to understanding long‐term host health and susceptibility to disease (Al Nabhani and Eberl 2020; Gensollen et al. 2016; Stiemsma and Turvey 2017).

## Author Contributions

**Abigail J. Miller:** conceptualization (equal), data curation (lead), formal analysis (lead), funding acquisition (equal), investigation (lead), methodology (equal), visualization (lead), writing – original draft (lead), writing – review and editing (equal). **Myung Chul Jo:** conceptualization (equal), investigation (equal), methodology (equal), writing – review and editing (equal). **Cassandra K. Hui:** formal analysis (equal), investigation (equal), visualization (equal), writing – review and editing (equal). **Juli Petereit:** formal analysis (equal), investigation (equal), methodology (equal), visualization (equal), writing – review and editing (equal). **Douglas C. Woodhams:** conceptualization (equal), funding acquisition (equal), methodology (equal), project administration (equal), supervision (equal), writing – review and editing (equal). **Jamie Voyles:** conceptualization (equal), funding acquisition (lead), methodology (equal), project administration (equal), resources (equal), supervision (lead), visualization (supporting), writing – original draft (supporting), writing – review and editing (equal).

## Funding

This research was funded by the National Institutes of Health (R15AI174147 to JV). We also received support from grants by the National Institute of General Medical Sciences (GM103440 and GM104944) from the National Institutes of Health.

## Ethics Statement

This research was approved by UNR IACUC (UNR no. 20‐12‐1114).

## Conflicts of Interest

The authors declare no conflicts of interest.

## Supporting information


**Figure S1:** A visual schematic of the experimental design.
**Figure S2:** Jaccard NMDS plot for *Xenopus tropicalis* treated with either an antimicrobial cocktail or a sham control solution and removed from sterile conditions at various time points during development. DNA was extracted from tadpoles 5 weeks after the embryos initially arrived in the laboratory.
**Figure S3:** Abundance by treatment group for families within the phylum Actinobacteriota measured 5 weeks after the embryos initially arrived in the laboratory.
**Figure S4:** Box plots showing (A) percent of antifungal bacteria in tadpole samples and (B) antifungal bacterial richness in tadpole samples, as calculated from the AmphiBac database. Each plot displays the median with upper and lower quartiles and maximum and minimum values for each experimental condition, ordered from most to least sterile exposure (*n* = 10).
**Figure S5:** Body condition (measured as mass/SVL) measured at 5, 7, and 11 weeks after *Xenopus tropicalis* embryos initially arrived in the laboratory. Each plot displays the median with upper and lower quartiles and maximum and minimum values for each experimental condition, ordered from most to least sterile exposure (*n* = 10).
**Table S2:** Post hoc pairwise analysis output for Jaccard PERMDISP analysis.

**Table S1:** File containing significantly different taxa identified from differential abundance analysis (ANCOM‐BC2).

## Data Availability

All the required data are uploaded to the Dryad repository accessible here: https://doi.org/10.5061/dryad.7wm37pw6x.

## References

[ece374063-bib-0001] Al Nabhani, Z. , and G. Eberl . 2020. “Imprinting of the Immune System by the Microbiota Early in Life.” Mucosal Immunology 13, no. 2: 183–189. 10.1038/s41385-020-0257-y. 31988466

[ece374063-bib-0002] Andersen, I. L. , E. Nævdal , and K. E. Bøe . 2011. “Maternal Investment, Sibling Competition, and Offspring Survival With Increasing Litter Size and Parity in Pigs ( *Sus scrofa* ).” Behavioral Ecology and Sociobiology 65, no. 6: 1159–1167. 10.1007/s00265-010-1128-4. 21743767 PMC3096772

[ece374063-bib-0003] Biberstein, E. L. , S. S. Jang , and D. C. Hirsh . 1985. “ *Nocardia asteroides* Infection in Horses: A Review.” Journal of American Veterinary Medical Association 186, no. 3: 273–277. 3882648

[ece374063-bib-0004] Bishop, T. F. , and C. W. Beck . 2021. “Bacterial Lipopolysaccharides Can Initiate Regeneration of the Xenopus Tadpole Tail.” iScience 24, no. 11: 103281. 10.1016/j.isci.2021.103281. 34765912 PMC8571501

[ece374063-bib-0005] Bletz, M. , and D. M. Woodhams . 2023. “AmphiBac Database [Internet].” https://github.com/AmphiBac/AmphiBac‐Database/tree/main/AmphiBac‐2023.2.

[ece374063-bib-0006] Busi, S. B. , L. De Nies , J. Habier , et al. 2021. “Persistence of Birth Mode‐Dependent Effects on Gut Microbiome Composition, Immune System Stimulation and Antimicrobial Resistance During the First Year of Life.” ISME Communications 1, no. 1: 8. 10.1038/s43705-021-00003-5. 36717704 PMC9723731

[ece374063-bib-0007] Campos‐Cerda, F. , and B. J. M. Bohannan . 2020. “The Nidobiome: A Framework for Understanding Microbiome Assembly in Neonates.” Trends in Ecology & Evolution 35, no. 7: 573–582. 10.1016/j.tree.2020.03.007. 32360079

[ece374063-bib-0008] Carotenuto, R. , M. M. Pallotta , M. Tussellino , and C. Fogliano . 2023. “ *Xenopus laevis* (Daudin, 1802) as a Model Organism for Bioscience: A Historic Review and Perspective.” Biology 12, no. 6: 890. 10.3390/biology12060890. 37372174 PMC10295250

[ece374063-bib-0009] Churgin, S. M. , J. L. L. Teng , J. H. P. Ho , et al. 2019. “First Case Report of Fatal *Nocardia nova* Infection in Yellow‐Bibbed Lory ( *Lorius chlorocercus* ) Identified by Multilocus Sequencing.” BMC Veterinary Research 15, no. 1: 4. 10.1186/s12917-018-1764-x. 30606196 PMC6318869

[ece374063-bib-0010] Cox, L. M. , S. Yamanishi , J. Sohn , et al. 2014. “Altering the Intestinal Microbiota During a Critical Developmental Window Has Lasting Metabolic Consequences.” Cell 158, no. 4: 705–721. 10.1016/j.cell.2014.05.052. 25126780 PMC4134513

[ece374063-bib-0011] Coyte, K. Z. , C. Rao , S. Rakoff‐Nahoum , and K. R. Foster . 2021. “Ecological Rules for the Assembly of Microbiome Communities.” PLoS Biology 19, no. 2: e3001116. 10.1371/journal.pbio.3001116. 33606675 PMC7946185

[ece374063-bib-0012] Du Pasquier, L. , J. Robert , M. Courtet , and R. Mußmann . 2000. “B‐Cell Development in the Amphibian *Xenopus* .” Immunological Reviews 175, no. 1: 201–213. 10.1111/j.1600-065X.2000.imr017501.x. 10933604

[ece374063-bib-0013] Edholm, E. S. 2018. “Flow Cytometric Analysis of *Xenopus* Immune Cells.” Cold Spring Harbor Protocols 2018, no. 7: pdb.prot097600. 10.1101/pdb.prot097600. 29669848

[ece374063-bib-0014] Emerson, K. J. , and S. K. Woodley . 2024. “Something in the Water: Aquatic Microbial Communities Influence the Larval Amphibian Gut Microbiota, Neurodevelopment and Behaviour.” Proceedings of the Royal Society B: Biological Sciences 291: 20232850. 10.1098/rspb.2023.2850. PMC10898966 38412968

[ece374063-bib-0015] Estaki, M. , L. Jiang , N. A. Bokulich , et al. 2020. “QIIME 2 Enables Comprehensive End‐To‐End Analysis of Diverse Microbiome Data and Comparative Studies With Publicly Available Data.” Current Protocols in Bioinformatics 70, no. 1: e100. 10.1002/cpbi.100. 32343490 PMC9285460

[ece374063-bib-0016] Fainsod, A. , and H. Kot‐Leibovich . 2018. “Xenopus Embryos to Study Fetal Alcohol Syndrome, a Model for Environmental Teratogenesis.” Biochemistry and Cell Biology 96, no. 2: 77–87. 29069552 10.1139/bcb-2017-0219

[ece374063-bib-0017] Gensollen, T. , S. S. Iyer , D. L. Kasper , and R. S. Blumberg . 2016. “How Colonization by Microbiota in Early Life Shapes the Immune System.” Science 352, no. 6285: 539–544. 10.1126/science.aad9378. 27126036 PMC5050524

[ece374063-bib-0018] Grogan, L. F. , J. Robert , L. Berger , et al. 2018. “Review of the Amphibian Immune Response to Chytridiomycosis, and Future Directions.” Frontiers in Immunology 9: 2536. 10.3389/fimmu.2018.02536. 30473694 PMC6237969

[ece374063-bib-0019] Huyck, R. W. , M. Nagarkar , N. Olsen , S. E. Clamons , and M. S. Saha . 2015. “Methylmercury Exposure During Early *Xenopus laevis* Development Affects Cell Proliferation and Death but Not Neural Progenitor Specification.” Neurotoxicology and Teratology 47: 102–113. 25496965 10.1016/j.ntt.2014.11.010 PMC4302163

[ece374063-bib-0020] Jani, A. J. , and C. J. Briggs . 2018. “Host and Aquatic Environment Shape the Amphibian Skin Microbiome but Effects on Downstream Resistance to the Pathogen Batrachochytrium Dendrobatidis Are Variable.” Frontiers in Microbiology 9: 487. 10.3389/fmicb.2018.00487. 29619014 PMC5871691

[ece374063-bib-0021] Jiménez, R. R. , A. Carfagno , L. Linhoff , et al. 2022. “Inhibitory Bacterial Diversity and Mucosome Function Differentiate Susceptibility of Appalachian Salamanders to Chytrid Fungal Infection.” Applied and Environmental Microbiology 88, no. 8: e01818‐21. 10.1128/aem.01818-21. 35348389 PMC9040618

[ece374063-bib-0022] Kim, B. R. , J. Shin , R. B. Guevarra , et al. 2017. “Deciphering Diversity Indices for a Better Understanding of Microbial Communities.” Journal of Microbiology and Biotechnology 27, no. 12: 2089–2093. 10.4014/jmb.1709.09027. 29032640

[ece374063-bib-0023] Knutie, S. A. , C. L. Wilkinson , K. D. Kohl , and J. R. Rohr . 2017. “Early‐Life Disruption of Amphibian Microbiota Decreases Later‐Life Resistance to Parasites.” Nature Communications 8, no. 1: 86. 10.1038/s41467-017-00119-0. PMC5519754 28729558

[ece374063-bib-0024] Kohl, K. D. , T. L. Cary , W. H. Karasov , and M. D. Dearing . 2013. “Restructuring of the Amphibian Gut Microbiota Through Metamorphosis.” Environmental Microbiology Reports 5, no. 6: 899–903. 10.1111/1758-2229.12092. 24249298

[ece374063-bib-0025] Konig, B. , and H. Markl . 1987. “Maternal Care in House Mice: I. The Weaning Strategy as a Means for Parental Manipulation of Offspring Quality.” Behavioral Ecology and Sociobiology 20, no. 1: 1–9. 10.1007/BF00292161.

[ece374063-bib-0026] Kuneš, J. , and J. Zicha . 2006. “Developmental Windows and Environment as Important Factors in the Expression of Genetic Information: A Cardiovascular Physiologist's View.” Clinical Science 111, no. 5: 295–305. 10.1042/CS20050271. 17034366

[ece374063-bib-0027] Lee, Y. , A. Williams , C. Hong , Y. You , M. Senoo , and J. Saint‐Jeannet . 2013. “Early Development of the Thymus in *Xenopus laevis* .” Developmental Dynamics 242, no. 2: 164–178. 10.1002/dvdy.23905. 23172757 PMC3640628

[ece374063-bib-0029] Lynn, M. A. , D. J. Tumes , J. M. Choo , et al. 2018. “Early‐Life Antibiotic‐Driven Dysbiosis Leads to Dysregulated Vaccine Immune Responses in Mice.” Cell Host & Microbe 23, no. 5: 653–660.e5. 10.1016/j.chom.2018.04.009. 29746836

[ece374063-bib-0030] Malik, R. , M. Krockenberger , C. O'Brien , et al. 2006. “ *Nocardia* Infections in Cats: A Retrospective Multi‐Institutional Study of 17 Cases.” Australian Veterinary Journal 84, no. 7: 235–245. 10.1111/j.1751-0813.2006.00004.x. 16879126

[ece374063-bib-0032] McGrath‐Blaser, S. , M. Steffen , T. U. Grafe , M. Torres‐Sánchez , D. S. McLeod , and C. R. Muletz‐Wolz . 2021. “Early Life Skin Microbial Trajectory as a Function of Vertical and Environmental Transmission in Bornean Foam‐Nesting Frogs.” Animal Microbiome 3, no. 1: 83. 10.1186/s42523-021-00147-8. 34930504 PMC8686334

[ece374063-bib-0033] Mehta, H. H. , and Y. Shamoo . 2020. “Pathogenic Nocardia: A Diverse Genus of Emerging Pathogens or Just Poorly Recognized?” PLoS Pathogens 16, no. 3: e1008280. 10.1371/journal.ppat.1008280. 32134995 PMC7058287

[ece374063-bib-0034] Miller, A. J. , J. Gass , M. C. Jo , et al. 2023. “Towards the Generation of Gnotobiotic Larvae as a Tool to Investigate the Influence of the Microbiome on the Development of the Amphibian Immune System.” Philosophical Transactions of the Royal Society, B: Biological Sciences 378: 20220125. 10.1098/rstb.2022.0125. PMC10258664 37305911

[ece374063-bib-0035] Miller, A. J. , and J. Voyles . 2026. “The Impact of Microbiome Diversity and Composition on Host Health and Susceptibility to Disease Across Amphibian Life Stages.” Frontiers in Amphibian and Reptile Science 3: 1666714. 10.3389/famrs.2025.1666714. PMC13580466 42751604

[ece374063-bib-0036] Myers, J. M. , J. P. Ramsey , A. L. Blackman , A. E. Nichols , K. P. C. Minbiole , and R. N. Harris . 2012. “Synergistic Inhibition of the Lethal Fungal Pathogen Batrachochytrium Dendrobatidis: The Combined Effect of Symbiotic Bacterial Metabolites and Antimicrobial Peptides of the Frog Rana muscosa.” Journal of Chemical Ecology 38, no. 8: 958–965. 10.1007/s10886-012-0170-2. 22914957

[ece374063-bib-0037] National Research Council. Committee on Care, and Use of Laboratory Animals . 1986. Guide for the Care and Use of Laboratory Animals. No. 86. US Department of Health and Human Services, Public Health Service, National Institutes of Health.

[ece374063-bib-0038] Nava‐González, B. , I. Suazo‐Ortuño , P. B. López , et al. 2021. “Inhibition of Batrachochytrium Dendrobatidis Infection by Skin Bacterial Communities in Wild Amphibian Populations.” Microbial Ecology 82, no. 3: 666–676. 10.1007/s00248-021-01706-x. 33598748

[ece374063-bib-0039] Nieuwkoop, P. D. , and J. P. Faber . 1994. Normal Table of *Xenopus laevis* (Daudin): A Systematical and Chronological Survey of the Development From the Fertilized Egg Till the End of Metamorphosis.

[ece374063-bib-0040] Olszak, T. , D. An , S. Zeissig , et al. 2012. “Microbial Exposure During Early Life Has Persistent Effects on Natural Killer T Cell Function.” Science 336, no. 6080: 489–493. 10.1126/science.1219328. 22442383 PMC3437652

[ece374063-bib-0041] Palmer, A. C. 2011. “Nutritionally Mediated Programming of the Developing Immune System.” Advances in Nutrition 2, no. 5: 377–395. 10.3945/an.111.000570. 22332080 PMC3183589

[ece374063-bib-0042] Quast, C. , E. Pruesse , P. Yilmaz , et al. 2012. “The SILVA Ribosomal RNA Gene Database Project: Improved Data Processing and Web‐Based Tools.” Nucleic Acids Research 41, no. D1: D590–D596. 10.1093/nar/gks1219. 23193283 PMC3531112

[ece374063-bib-0043] R Core Team . n.d. R: A Language and Environment for Statistical Computing. R Foundation for Statistical Computing. https://www.R‐project.org/.

[ece374063-bib-0044] Rawstern, A. H. , D. J. Hernandez , and M. E. Afkhami . 2025. “Central Taxa Are Keystone Microbes During Early Succession.” Ecology Letters 28, no. 1: e70031. 10.1111/ele.70031. 39737770 PMC11687413

[ece374063-bib-0045] Rendueles, O. , L. Ferrières , M. Frétaud , et al. 2012. “A New Zebrafish Model of Oro‐Intestinal Pathogen Colonization Reveals a Key Role for Adhesion in Protection by Probiotic Bacteria.” PLoS Pathogens 8, no. 7: e1002815. 22911651 10.1371/journal.ppat.1002815 PMC3406073

[ece374063-bib-0046] Retnaningdyah, C. , and D. Ebert . 2016. “Bleach Solution Requirement for Hatching of *Daphnia magna* Resting Eggs.” Journal of Tropical Life Science 6, no. 3: 93129.

[ece374063-bib-0047] Ribeiro, M. G. , T. Salerno , A. L. D. Mattos‐Guaraldi , et al. 2008. “Nocardiosis: An Overview and Additional Report of 28 Cases in Cattle and Dogs.” Revista do Instituto de Medicina Tropical de São Paulo 50, no. 3: 177–185. 10.1590/S0036-46652008005000004. 18516465

[ece374063-bib-0048] Robert, J. , and Y. Ohta . 2009. “Comparative and Developmental Study of the Immune System in *Xenopus* .” Developmental Dynamics 238, no. 6: 1249–1270. 10.1002/dvdy.21891. 19253402 PMC2892269

[ece374063-bib-0049] Rollins‐Smith, L. A. 1998. “Metamorphosis and the Amphibian Immune System.” Immunological Reviews 166, no. 1: 221–230. 10.1111/j.1600-065x.1998.tb01265.x. 9914915

[ece374063-bib-0050] Rollins‐Smith, L. A. , D. A. Parker Needham , A. T. Davis , and P. J. Blair . 1996. “Late Thymectomy in Xenopus Tadpoles Reveals a Population of t Cells That Persists Through Metamorphosis.” Developmental and Comparative Immunology 20, no. 3: 165–174. 10.1016/0145-305X(96)00018-3. 8955591

[ece374063-bib-0051] Schuck, L. K. , W. J. Neely , S. M. Buttimer , et al. 2024. “Effects of Grassland Controlled Burning on Symbiotic Skin Microbes in Neotropical Amphibians.” Scientific Reports 14, no. 1: 959. 10.1038/s41598-023-50394-9. 38200064 PMC10781984

[ece374063-bib-0052] Selevan, S. G. , C. A. KimmelA , and P. Mendola . 2000. “Identifying Critical Windows of Exposure for Children's Health.” Environmental Health Perspectives 108. 10.1289/ehp.00108s3451 PMC1637810 10852844

[ece374063-bib-0053] Stiemsma, L. T. , and S. E. Turvey . 2017. “Asthma and the Microbiome: Defining the Critical Window in Early Life.” Allergy, Asthma and Clinical Immunology 13, no. 1: 3. 10.1186/s13223-016-0173-6. PMC5217603 28077947

[ece374063-bib-0054] Tobias, M. L. , S. S. Viswanathan , and D. B. Kelley . 1998. “Rapping, a Female Receptive Call, Initiates Male–Female Duets in the South African Clawed Frog.” Proceedings of the National Academy of Sciences 95, no. 4: 1870–1875. 10.1073/pnas.95.4.1870. PMC19205 9465109

[ece374063-bib-0055] Vismara, C. , R. Bacchetta , A. Di Muzio , et al. 2006. “H_2_O_2_ Induces Abnormal Tail Flexure in Xenopus Embryos: Similarities With Paraquat Teratogenic Effects.” Birth Defects Research Part B: Developmental and Reproductive Toxicology 77, no. 3: 238–243. 16767755 10.1002/bdrb.20080

[ece374063-bib-0056] Warne, R. W. , L. Kirschman , and L. Zeglin . 2019. “Manipulation of Gut Microbiota During Critical Developmental Windows Affects Host Physiological Performance and Disease Susceptibility Across Ontogeny.” Journal of Animal Ecology 88, no. 6: 845–856. 10.1111/1365-2656.12973. 30828805

[ece374063-bib-0057] Wlizla, M. , S. McNamara , and M. E. Horb . 2018. “Generation and Care of Xenopus Laevis and *Xenopus tropicalis* Embryos.” In Xenopus: Methods and Protocols, 19–32. Springer New York. 10.1007/978-1-4939-8784-9_2 PMC6396978 30151756

[ece374063-bib-0058] Woodhams, D. C. , R. A. Alford , R. E. Antwis , et al. 2015. “Antifungal Isolates Database of Amphibian Skin‐Associated Bacteria and Function Against Emerging Fungal Pathogens: Ecological Archives E096‐059.” Ecology 96, no. 2: 595. 10.1890/14-1837.1.

[ece374063-bib-0059] Woodhams, D. C. , J. McCartney , J. B. Walke , and R. Whetstone . 2023. “The Adaptive Microbiome Hypothesis and Immune Interactions in Amphibian Mucus.” Developmental and Comparative Immunology 145: 104690. 10.1016/j.dci.2023.104690. 37001710 PMC10249470

[ece374063-bib-0060] Zahn, N. , C. James‐Zorn , V. G. Ponferrada , et al. 2022. “Normal Table of *Xenopus* Development: A New Graphical Resource.” Development 149, no. 14: dev200356. 10.1242/dev.200356. 35833709 PMC9445888

