# Supplementary material for: Effects of Timing of Microbial Exposure on Microbiome Assembly and Amphibian Immune Development
Source: Ecol Evol. 2026 Aug 2;16(8):e74063. doi: 10.1002/ece3.74063 (PMC13429884; doi:10.1002/ece3.74063)
Supplement: Supplementary file 1 — Figure S1: A visual schematic of the experimental design. Figure S2: Jaccard NMDS plot for Xenopus tropicalis treated with either an antimicrobial cocktail or a sham control solution and removed from sterile conditions at various time points during development. DNA was extracted from tadpoles 5 weeks after the embryos initially arrived in the laboratory. Figure S3: Abundance by treatment group for families within the phylum Actinobacteriota measured 5 weeks after the embryos initially arrived in the laboratory. Figure S4: Box plots showing (A) percent of antifungal bacteria in tadpole samples and (B) antifungal bacterial richness in tadpole samples, as calculated from the AmphiBac database. Each plot displays the median with upper and lower quartiles and maximum and minimum values for each experimental condition, ordered from most to least sterile exposure (n = 10). Figure S5: Body condition (measured as mass/SVL) measured at 5, 7, and 11 weeks after Xenopus tropicalis embryos initially arrived in the laboratory. Each plot displays the median with upper and lower quartiles and maximum and minimum values for each experimental condition, ordered from most to least sterile exposure (n = 10). Table S2: Post hoc pairwise analysis output for Jaccard PERMDISP analysis. [file ECE3-16-e74063-s002.pdf]

Appendix S1

Supplemental Figures for:

Effects of timing of microbial exposure on microbiome assembly and amphibian immune development

Authors: Abigail J. Miller[1], Myung Chul Jo[2], Cassandra K. Hui[3], Juli Petereit[3], Douglas C. Woodhams[4], Jamie Voyles[1]

Affiliations

1. Department of Biology, University of Nevada, Reno, Reno NV, USA
2. Environmental Health and Safety, University of Nevada, Reno, Reno NV, USA
3. Nevada Bioinformatics Center, University of Nevada, Reno, Reno NV, USA
4. Department of Biology, University of Massachusetts Boston, Boston, MA, USA

This file includes:

Figure descriptions

Fig. S1-S4

Fig S1. A visual schematic of the experimental design.

Fig S2. Jaccard NMDS plot for *Xenopus tropicalis* treated with either an antimicrobial cocktail or a sham control solution and removed from sterile conditions at various time points during development. DNA was extracted from tadpoles five weeks after the embryos initially arrived in the laboratory.

Fig S3. Abundance by treatment group for families within the phylum Actinobateriota measured five weeks after the embryos initially arrived in the laboratory.

Fig S4. Box plots showing (A) percent of antifungal bacteria in tadpole samples and (B) antifungal bacterial richness in tadpole samples, as calculated from the AmphiBac database. Each plot displays the median with upper and lower quartiles and maximum and minimum values for each experimental condition, ordered from most to least sterile exposure (n = 10).

Fig S5. Body condition (measured as mass/SVL) measured at 5 weeks, 7 weeks, and 11 weeks after *Xenopus tropicalis* embryos initially arrived in the laboratory. Each plot displays the median with upper and lower quartiles and maximum and minimum values for each experimental condition, ordered from most to least sterile exposure (n = 10).

Table S2. Post-hoc pairwise analysis output for Jaccard PERMDISP analysis.

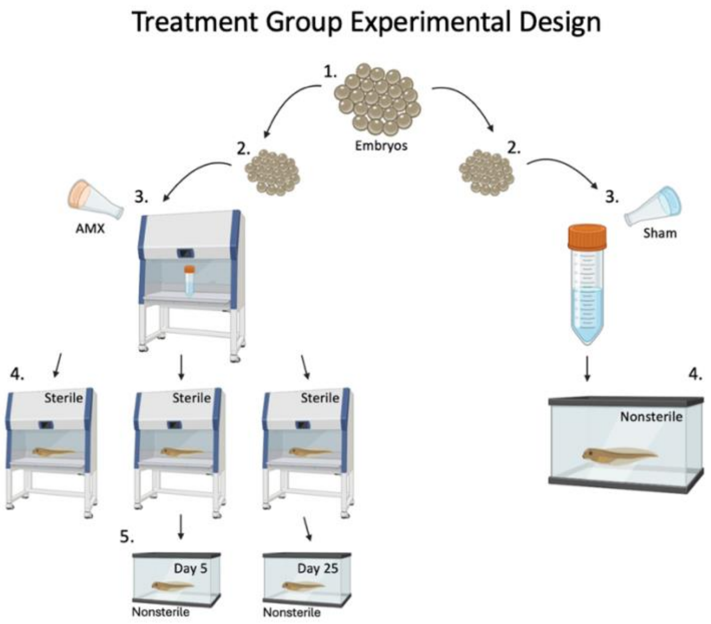

Fig. S1

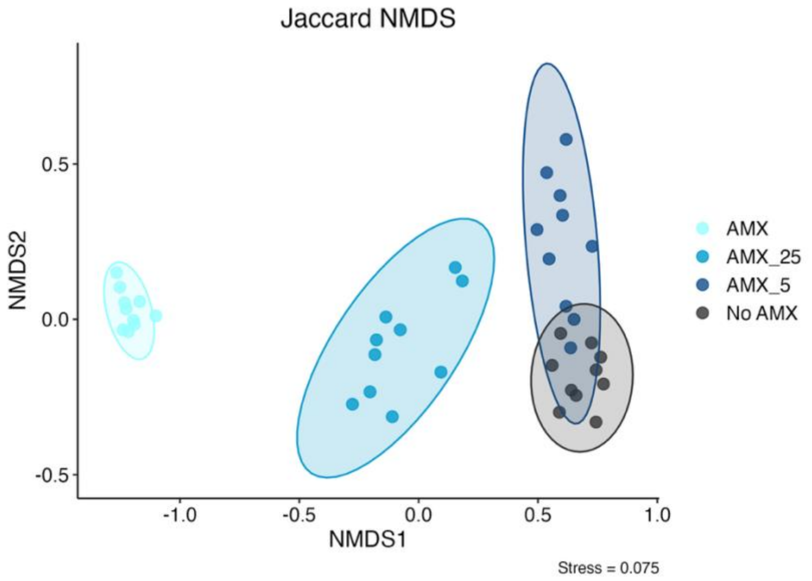
Jaccard NMDS
NMDS2
NMDS1
0.5
0.0
-0.5
-1.0
-0.5
0.0
0.5
1.0
AMX
AMX_25
AMX_5
No AMX
Stress = 0.075

Fig. S2

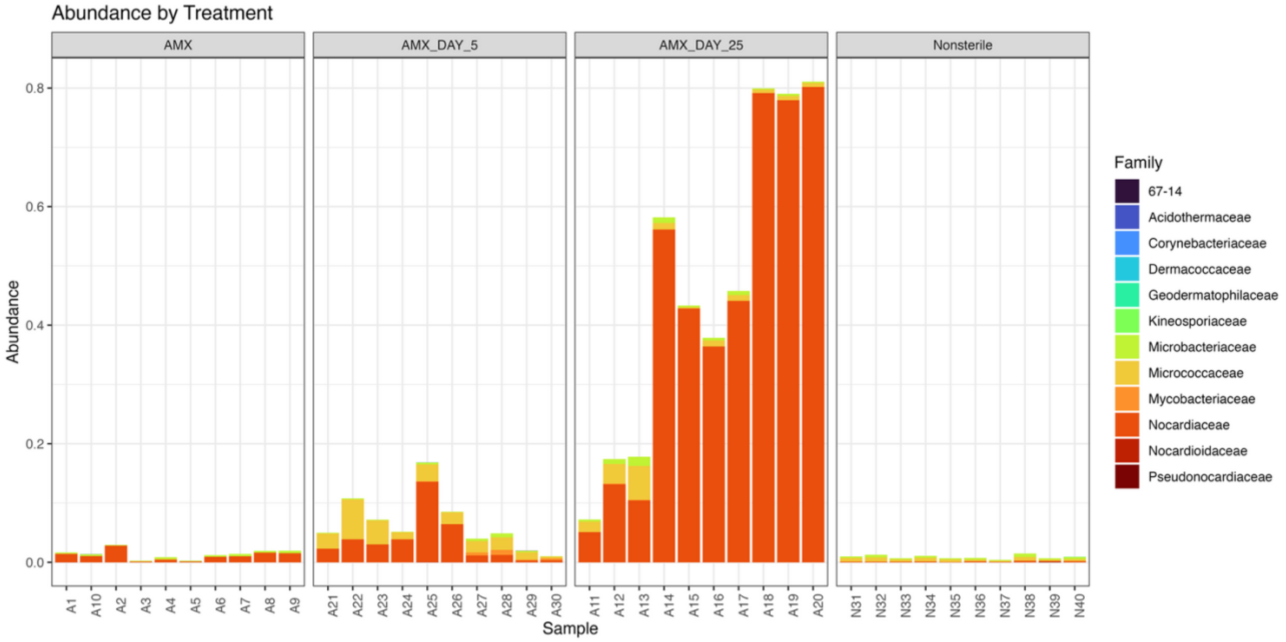
Abundance by Treatment
AMX
AMX_DAY_5
AMX_DAY_25
Nonsterile
Abundance
0.0
0.2
0.4
0.6
0.8
Sample
A1 A10 A2 A3 A4 A5 A6 A7 A8 A9
A21 A22 A23 A24 A25 A26 A27 A28 A29 A30
A11 A12 A13 A14 A15 A16 A17 A18 A19 A20
N31 N32 N33 N34 N35 N36 N37 N38 N39 N40
Family
67-14
Acidothermaceae
Corynebacteriaceae
Dermacoccaceae
Geodermatophilaceae
Kineosporiaceae
Microbacteriaceae
Micrococcaceae
Mycobacteriaceae
Nocardiaceae
Nocardioidaceae
Pseudonocardiaceae

Fig. S3

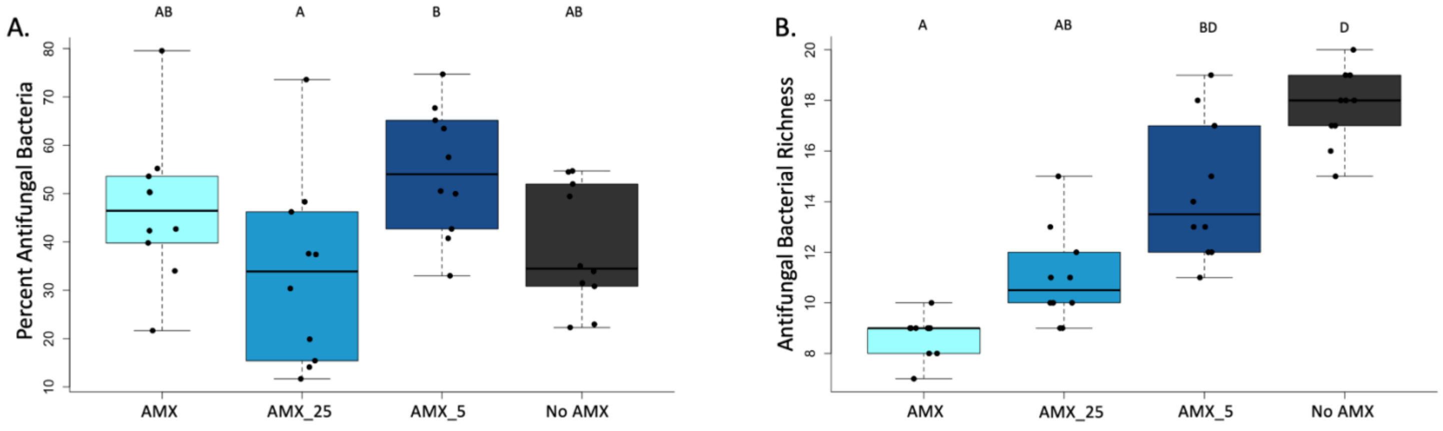
A.
AB
A
B
AB
Percent Antifungal Bacteria
10
20
30
40
50
60
70
80
AMX
AMX_25
AMX_5
No AMX
B.
A
AB
BD
D
Antifungal Bacterial Richness
8
10
12
14
16
18
20
AMX
AMX_25
AMX_5
No AMX

Fig. S4

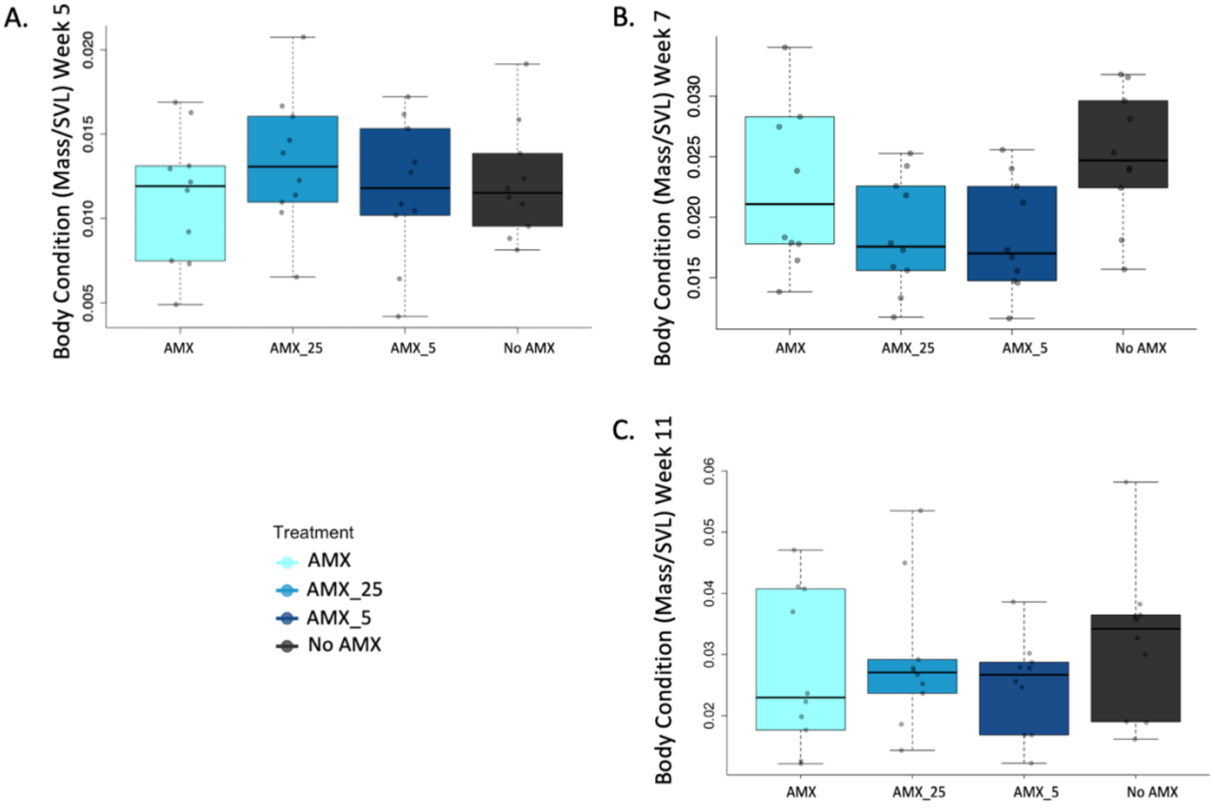
A.
Body Condition (Mass/SVL) Week 5
0.005
0.010
0.015
0.020
AMX
AMX_25
AMX_5
No AMX
B.
Body Condition (Mass/SVL) Week 7
0.015
0.020
0.025
0.030
AMX
AMX_25
AMX_5
No AMX
C.
Body Condition (Mass/SVL) Week 11
0.02
0.03
0.04
0.05
0.06
AMX
AMX_25
AMX_5
No AMX
Treatment
AMX
AMX_25
AMX_5
No AMX

Fig. S5

Pairwise_PERMDISP_Jaccard

diff	lwr	upr	p adj	Comparison
**0.107470473431108**	0.0503326265692915	0.164608320292925	7.02390772324701E-05	AMX_25-AMX
**0.114482999574052**	0.0573451527122349	0.171620846435869	2.56783192060794E-05	AMX_5-AMX
**0.0656472172102681**	0.00850937034845115	0.122785064072085	0.0190306341816548	No AMX-AMX
**0.00701252614294345**	-0.0501253207188735	0.0641503730047604	0.987332193399174	AMX_5-AMX_25
**-0.0418232562208403**	-0.0989611030826572	0.0153145906409766	0.217628651961723	No AMX-AMX_25
**-0.0488357823637838**	-0.105973629225601	0.00830206449803313	0.116506757134196	No AMX-AMX_5
